# Superiority of tenofovir alafenamide fumarate over entecavir for serum HBsAg level reduction in patients with chronic HBV infection: A 144-week outcome study after switching of the nucleos(t)ide analog

**DOI:** 10.1371/journal.pone.0262764

**Published:** 2022-02-18

**Authors:** Yoshihito Uchida, Masamitsu Nakao, Shunsuke Yamada, Shohei Tsuji, Hayato Uemura, Jun-ichi Kouyama, Kayoko Naiki, Kayoko Sugawara, Nobuaki Nakayama, Yukinori Imai, Tomoaki Tomiya, Satoshi Mochida

**Affiliations:** Faculty of Medicine, Department of Gastroenterology & Hepatology, Saitama Medical University, Saitama, Japan; Kaohsiung Medical University Hospital, TAIWAN

## Abstract

**Background:**

To evaluate the long-term efficacy of switching of the nucleos(t)ide analog used for treatment from entecavir (ETV) to tenofovir alafenamide fumarate (TAF) in patients with chronic HBV infection.

**Methods:**

A total of 103 patients with serum HBsAg levels of ≥100 IU/mL who had received ETV were enrolled. The nucleos(t)ide analog used for the treatment was switched from ETV to TAF, and the changes in serum HBsAg levels during the 144-week period before and after the drug switching were compared in 74 patients who had received ETV at least for 192 weeks.

**Results:**

Significant decreases of serum HBsAg levels were observed during both the ETV and the TAF administration period, although the degree of reduction was greater during the latter period than during the former period (P<0.001). Significant decreases of serum HBsAg levels were seen in both patients with genotype B HBV infection and genotype C HBV infection, irrespective of the serum HBsAg and HBcrAg levels at the time of the drug switching.

**Conclusion:**

Switching of the nucleos(t)ide analog used for treatment from ETV to TAF merits consideration in patients with chronic HBV infection, since the extent of reduction of the serum HBsAg level was greater during the TAF treatment period than during the ETV treatment period.

## Introduction

According to the Cancer Information Service of the National Cancer Center in Japan [1], Japanese patients dying of hepatocellular carcinoma (HCC) have been decreasing year-on-year after 2003. The percentage of patients with hepatitis B virus (HBV) infection among patients with HCC, however, has not decreased in Japan, while that of patients with hepatitis C virus (HCV) infection have been decreasing [2]. To minimize the risk of HCC development in patients with chronic HBV infection, efforts should be made to achieve reduction of both the serum HBV-DNA and serum HBsAg levels [3,4]. Study groups from Taiwan have reported that serum HBsAg levels of <1,000 IU/mL [4] and serum HBV-DNA levels of <2,000 IU/mL [3] are required to prevent the development of HCC in patients with chronic HBV infection. Thus, the Japan Society of Hepatology (JSH) proposed the following treatment goals for minimizing the risk of development of HCC in patients with chronic HBV infection; short-term goal: to achieve undetectable serum HBV-DNA levels accompanied by sustained normal serum ALT levels; long-term treatment goal: to achieve undetectable serum HBsAg [5].

Nucleos(t)ide analogs are useful to achieve the aforementioned short-term treatment goal in patients with chronic HBV infection, while pegylated-interferon (Peg-IFN) monotherapy or add-on Peg-IFN therapy with nucleos(t)ide analogs (hereafter, add-on Peg-IFN therapy) is known to allow the long-term treatment goal of reduction of the serum HBsAg levels to be achieved [6]. Peg-IFN therapy is, however, unsuitable in the majority of patients because of the high incidence of adverse events and the inconvenience of weekly subcutaneous injections for at least 48 weeks. Therefore, in Japan, either entecavir (ETV), a nucleoside analog of guanine, or tenofovir disoproxil fumarate (TDF)/tenofovir alafenamide fumarate (TAF), a nucleotide analog of adenine, has been used as the first-line antiviral agent for the treatment of chronic HBV infection [5]. Although the extent of reduction of the serum HBsAg level obtained with these nucleos(t)ide analogs is smaller than that obtained with Peg-IFN monotherapy or add-on Peg-IFN therapy [6], a greater degree of reduction of the serum HBsAg levels has been noted in patients receiving TDF than in those receiving ETV [7]. Thus, TDF may be superior to ETV for minimizing the risk of HCC development. TDF, however, cannot utilized for prolonged periods of time in patients, since tenofovir, derived from TDF following intestinal absorption, may cause renal tubular damage, leading to the development of hypophosphatemia, renal impairment, and bone mineral deficiency [8–10]. Although these adverse events have not yet been known to occur in patients receiving TAF [8–10], which is a prodrug converted to tenofovir exclusively in the hepatocytes, the antiviral effects of TAF, especially in relation to its efficacy of reducing the serum HBsAg level in patients with chronic HBV infection, still remain to be precisely elucidated.

Thus, we previously conducted a prospective clinical study to compare the antiviral effect, in terms of decreasing the serum HBsAg level, between ETV and TAF in patients with chronic HBV infection, after switching the nucleos(t)ide analog used for treatment from ETV to TAF, and revealed the superiority of TAF over ETV in the short term, over an observation period of 48 weeks after the drug switching [11]. In that study, the extent of reduction of the HBsAg level during the 48-week TAF treatment period was higher than that during a 48-week ETV treatment period, especially in patients without underlying cirrhosis, patients with genotype B HBV infection, and patients with serum HBcrAg levels of <3.0 Log U/mL [11]. In the present study, the same cohort was followed up further for 144 weeks, and antiviral efficacy and safety of TAF over the long term, that is, 144 weeks after the switching of the nucleos(t)ide analog from ETV to TAF were evaluated, by comparing the relevant parameters between a 144-week treatment period with ETV prior to the drug switching and 144-week treatment period with TAF after the drug switching, in patients who had received ETV at least for 192 weeks before the drug switching.

## Patients and methods

### Patients and study design

This was a single-arm prospective observational study conducted in patients of Saitama Medical University Hospital (Moroyama, Saitama, Japan). The patients with chronic HBV infection who were receiving ETV monotherapy were enrolled in this study; the patients were taking oral ETV (Baraclude^®^; Bristol-Myers Squibb, New York, NY, US) at the dose of 0.5 mg once a day. In all patients, the antiviral agent was switched from oral ETV to oral TAF (VEMLIDY^®^; Gilead Sciences, Inc., Foster City, CA, US) at 25 mg once a day.

All the patients were enrolled between 2^nd^ March 2018 and 25^th^ May 2018; the eligibility criteria were as follows: 1) ≥20 years of age; 2) serum HBsAg level of ≥100 IU/mL; 3) received ETV treatment for longer than 48 weeks at the time of drug switching; 3) availability of sufficient clinical information about the patient prior to the drug switching. Patients having coinfection with either HCV or human immunodeficiency virus (HIV), those receiving immunosuppressive agents, and those with underlying decompensated cirrhosis and/or end-stage HCC, were excluded from the study.

The nucleos(t)ide analog used for the treatment was switched from ETV to TAF, and the changes in serum HBsAg levels and laboratory data during the 144-week period before the drug switching were compared with those during the 144-week period after the drug switching retrospectively in the similar patients who had received ETV at least for 192 weeks.

Written informed consent was obtained from each of the patients. The study conformed to the ethical guidelines laid down in the Declaration of Helsinki and was conducted with the approval of the Institutional Review Board of Saitama Medical University Hospital (17–132); the study was also registered with the University Hospital Medical Information Network Clinical Trials Registry (UMIN-CTR) as UMIN000030661.

### Evaluation of the Virologic Markers of HBV

Serum HBV-DNA levels were measured with COBAS TaqMan HBV Test v2.0 (Roche Diagnostics K.K., Tokyo, Japan); the lower limit of quantification was 20 IU/mL. Serum HBsAg levels were measured by chemiluminescence immunoassay using the Architect HBsAg QT assay kit (Abbott Japan Corp, Tokyo, Japan), and the HBeAg, anti-HBe and HBcrAg levels were measured by chemiluminescence enzyme immunoassay using the Architect HBeAg (Abbott Japan Corp,), Architect HBeAb (Abbott Japan Corp,) and Lumipulse HBcrAg (Fuji-Rebio, Tokyo, Japan) kits [12], respectively. The infecting HBV genotype was identified by enzyme-linked immunosorbent assay using monoclonal antibodies directed against type-specific epitopes in the preS2-region (Institute of Immunology, Tokyo, Japan).

### Therapeutic efficacy of the nucleos(t)ide analogs

Laboratory parameters, including serum HBV markers, liver function tests, and fasting serum levels of creatinine, total cholesterol and triglyceride were prospectively examined at the time of the drug switching, and at 8, 24, 48, 72, 96, and 144 weeks after the switching. At each point, the eGFR was calculated using the following formula [13]; for men: eGFR (mL/min/1.73 m^2^) = 194 × serum creatinine level (sCr)^−1.094^ × age^−0.287^; for women: eGFR = 194 × sCr^−1.094^ × age^−0.287^ × 0.739. The antiviral efficacy and safety of TAF during the 144-week TAF treatment period in the patients were retrospectively compared with those of ETV during a 144-week ETV treatment period prior to the drug switching.

Moreover, the therapeutic efficacies of the nucleos(t)ide analogs were determined based on the change of the serum HBsAg levels during a 144-week treatment period with the respective drugs: increase and decrease of the level by ≥0.24 Log IU/mL was defined as “increased” and “decreased,” respectively, and increase or decrease by <0.24 Log IU/mL was defined as “unchanged.”, since previous observations in a large cohort by Suzuki et al. revealed that the mean annual reduction of the serum HBsAg level during ETV monotherapy was 0.08 Log IU/mL in previously nucleos(t)ide analog-naïve patients with HBV infection [14]. Patients in whom the serum HBsAg levels increased/remained unchanged during the ETV administration period, but decreased during the TAF administration period were labeled as “TAF responders.” In addition, patients in whom the serum HBsAg levels decreased during the ETV administration period, but decreased even further during the TAF administration period by at least 0.24 Log IU/mL were also labeled as “TAF responders”.

### Statistical analysis

Categorical data were compared by Fisher’s exact test. Distributions of the continuous variables were analyzed by Mann–Whitney’s *U*-test. Differences in continuous variables were assessed by Wilcoxon’s signed-rank test. The propensity score matching model was estimated using a logistic regression model with adjustments for patients characteristics such as the age, sex, infecting HBV genotype, presence/absence of underlying cirrhosis, HBsAg and HBcrAg levels, and HBeAg positivity/negativity. Changes of the serum HBsAg levels during the ETV administration period and TAF administration period were compared using the McNemar test. Factors associated with the therapeutic efficacy of TAF were also evaluated by multivariate logistic regression analysis. All tests of significance were two-tailed, and P values of less than 0.05 were considered as denoting statistical significance. SPSS Statistics version 27 (IBM SPSS, Tokyo, Japan) was used for the analyses.

## Results

### Demographic characteristics and clinical features of the patients

**Fig 1:**
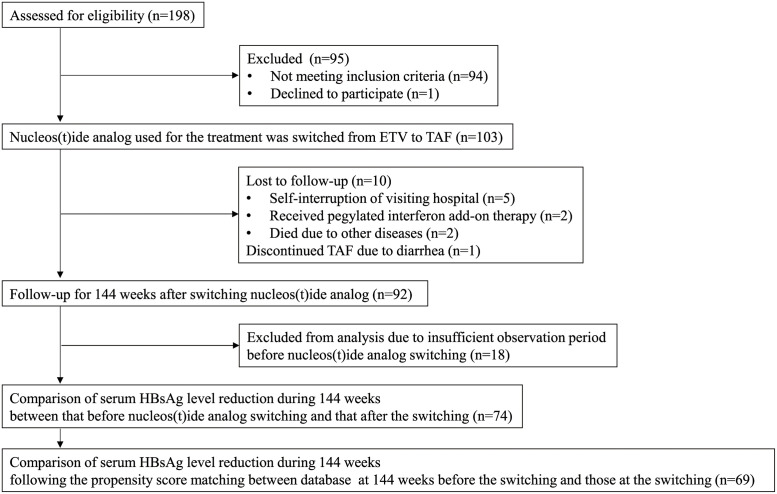
Flow chart of the study population.

A total of 198 patients with chronic hepatitis B infection were screened for eligibility and 103 (52.0%) patients were enrolled, and nucleos(t)ide analog used for the treatment was switched from ETV to TAF. Among these patients, 10 patients were lost to follow up: due to self-interruption of visiting hospital in 5 patients, receiving Peg-IFN add-on therapy following the drug switching in 2 patients, dead due to other diseases in 2 patients. One patient discontinued TAF due to diarrhea. Consequently, 92 patients received TAF at least for 144weeks after the drug switching, and the changes in serum HBsAg levels and laboratory data during the 144-week period before and after the drug switching were compared in 74 patients who had received ETV at least for 192 weeks (Fig 1).

The demographic characteristics and clinical features of the 74 patients at 144-week prior to the drug switching, at the time of switching of the nucleos(t)ide analog from ETV to TAF, at 144-week after drug switching are summarized in Table 1. At the timing of drug switching. None of the patients had previously received IFN-based antiviral therapies, lamivudine (LAM), adefovir dipivoxil (ADV) or TDF, while 3 patients (4.1%) were receiving lipid-lowering drugs. The median period of ETV administration was 7.7 years (IQR, 5.4 to 9.2 years). The infecting HBV genotypes were A, B, and C in 2 (2.7%), 18 (24.3%), and 54 patients (73.0%), respectively, and the serum HBV-DNA levels were less than 20 IU/mL in 73 patients (98.6%), and 40 IU/mL in the remaining patient with genotype C HBV infection. The median serum HBsAg level (IU/mL) was 1,104 (IQR, 551 to 2,561), and the serum HBsAg levels (IU/mL) were <10^3^ in 36 patients (48.6%), 10^3^ to 10^4^ in 31 patients (41.9%), and ≥10^4^ in 7 patients (9.5%). The test for serum HBeAg was positive in 8 patients (10.8%), while serum HBcrAg was undetectable (<3.0 Log U/mL) in 39 patients (52.7%) and detectable in 29 patients (39.2%); the test result was indeterminate in 6 patients (8.1%).

**Table 1:** Demographic characteristics and clinical features of the 74 patients with HBV chronic infection enrolled in the study.

Factors	Median (Interquartile Range) or Number of Patients (%)			P-values (At 144-week prior to the drug switching vs at the time of the drug switching)	P-values (At the time of the drug switching vs at 144-week after drug switching
	At 144-week prior to the drug switching	At the time of the drug switching	At 144-week after drug switching		
Age: years old	58 (48–65)	61 (51–68)	64 (54–71)	<0.001	
Male/Female	—	40 (54.1)/34 (45.9)	—	—	
Underling cirrhosis: present	5 (6.8)	5 (6.8)	5 (6.8)	1.000	
Previously treated HCC: present	6 (8.1)	8 (10.8)	8 (10.8)	0.500	1.000
Antihyperlipidemic drug: present	3 (4.1)	3 (4.1)	3 (4.1)	1.000	1.000
Period of ETV administration: years	4.7 (2.4–6.2)	7.7 (5.4–9.2)	—	0.005	—
Genotype A/B/C	—	2 (2.7)/18 (24.3)/54 (73.0)	—	—	—
HBV-DNA: IU/mL <20/20≦	73 (98.6)/1 (1.4)	73 (98.6)/1 (1.4)	74 (100)/0 (0)	1.000	1.000
HBsAg: IU/mL <10^2^/10^2^≦<10^3^/10^3^≦<10^4^/10^4^≦	1,185 (618–2947) 0 (0)/33 (44.6)/34 (45.9)/7 (9.5)	1,104 (551–2,561) 0 (0)/36 (48.6)/31 (41.9)/7 (9.5)	830 (215–2,021) 6 (8.1)/38 (51.4)/27 (36.5)/3 (4.1)	0.008 0.453	<0.001 0.002
HBeAg: positive	11 (14.9)	8 (10.8)	6 (8.1)	0.250	0.244
HBcrAg: Log U/mL <3.0/≧3.0/indeterminate	36 (48.6)/29 (39.2)/9 (12.2)	39 (52.7)/29 (39.2)/6 (8.1)	45 (60.8)/24 (32.4)/5 (6.8)	1.000	0.656
Platelets: 10^3^/mm^3^	203 (163–243)	197 (166–256)	213(186–250)	0.506	
AST: U/L	22 (19–27)	22 (19–24)	23 (20–27)	0.997	0.350
ALT: U/L	16 (13–22)	17 (13–20)	17 (13–24)	0.783	0.295
Creatinine: mg/dL	0.68 (0.63–0.80)	0.70 (0.62–0.81)	0.75 (0.65–0.81)	0.181	0.021
eGFR: mL/min/1.73 m^2^	77.8 (68.9–88.5)	75.4 (69.5–83.6)	75.8 (64.8–82.4)	0.034	0.002
Inorganic phosphorus: mg/dL	—	3.3 (2.9–3.8)	3.4 (3.0–3.7)	—	0.429
Total cholesterol: mg/dL	192 (167–216)	194 (169–222)	194 (175–233)	0.038	0.021
Triglyceride: mg/dL	83 (60–114)	88 (62–123)	96 (69–127)	0.044	0.001

HCC, hepatocellular carcinoma; ETV, entecavir; HBsAg, hepatitis B surface-antigen; HBeAg, hepatitis B e-antigen; HBcrAg, hepatitis B core-related-antigen; AST, aspartate aminotransferase; ALT, alanine aminotransferase; eGFR, estimate glomerular filtration rate.

### Virologic Markers in the 74 patients who had received ETV for at least 198 weeks in whom the nucleos(t)ide analog was switched from ETV to TAF

The serum HBV-DNA levels decreased to below 20 IU/mL within 48 weeks of switching of the nucleos(t)ide analog in the patient in whom the level was 40 IU/mL at the time of the switching. No viral breakthrough was seen in any of the patients after the switching. Among the 8 patients who were seropositive for HBeAg during the ETV treatment period, two became seronegative for serum HBeAg within 48 weeks of switching of the drug from ETV to TAF.

**Fig 2:**
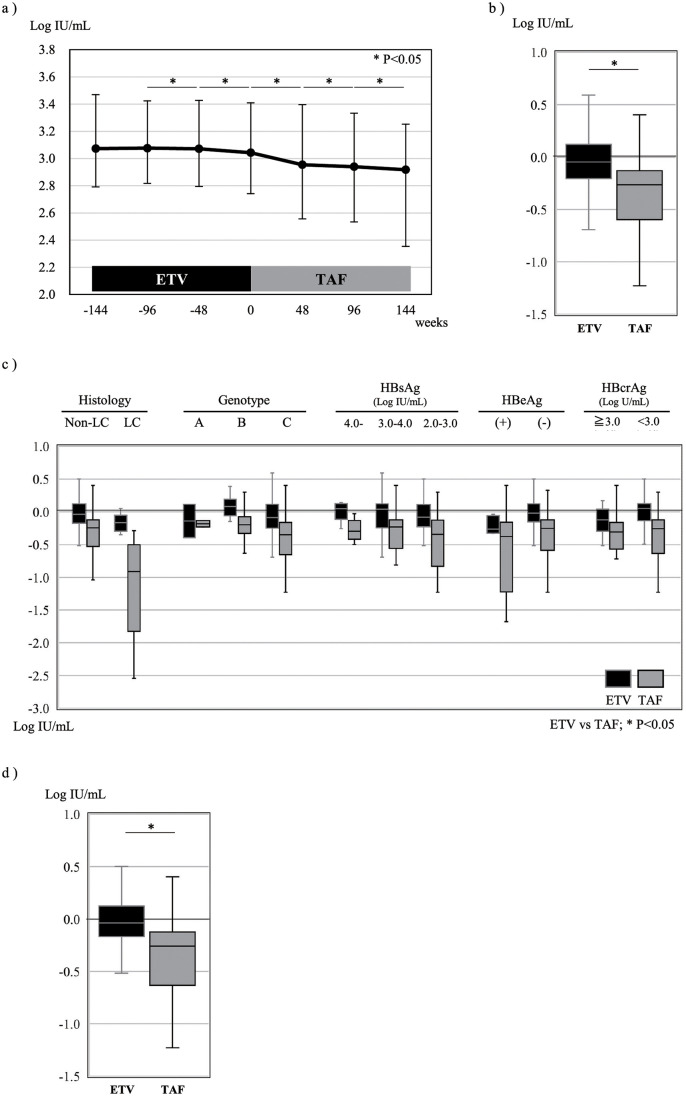
Serum HBsAg levels in patients with hepatitis B virus (HBV) infection before and after switching of the nucleos(t)ide analog used for treatment from entecavir (ETV) to tenofovir alafenamide (TAF). a) Serum HBsAg levels; the points and error bars represent the median values and quartiles (Q1 and Q3), respectively. b-d) The box and whisker represent the quartiles (Q1 and Q3) and range, respectively. b) Median reduction of the serum HBsAg level during the 144-week treatment periods with ETV and TAF. c) Median reduction of the serum HBsAg level during the ETV and TAF treatment periods depending on the virologic and clinical features of the patients. LC: Liver cirrhosis; HBeAg: Hepatitis B e antigen; HBcrAg: Hepatitis B core-related antigen. d) Median reduction of the serum HBsAg level during the 144-week ETV and TAF treatment periods following propensity score matching of the patients for their demographic characteristics and clinical features at 144 weeks before the switching and those at the time of switching.

The median serum HBsAg levels (IU/mL) were 1,186 (3.07), 1,191 (3.08), and 1,180 (3.07) at 144, 96 and 48 weeks prior to the drug switching, 1,104 (3.04) at the time of the switching, and 889 (2.95), 871 (2.94), and 828 (2.92) at 48, 96 and 144 weeks after the switching, respectively. Thus, significant decrease of the serum HBsAg levels was observed both during the 144-week ETV treatment period prior to the drug switching (P<0.001) and 144-week TAF treatment period after the drug switching (P<0.001) (Fig 2a) while serum HBsAg levels became undetectable in none of patients during the 144-week TAF treatment period. The degrees of decrease of the serum HBsAg levels were, however, greater during the TAF treatment period than during the ETV treatment period; the serum HBsAg level decreased by a median (Log IU/mL/144 weeks) of -0.261 (IQR, -0.599 to -0.130) during the TAF treatment period, as compared to the corresponding value of -0.046 (IQR, -0.205 to +0.122) during the ETV treatment period (P<0.001) (Fig 2b). Comparison of the degrees of decrease of the serum HBsAg levels among subgroups classified by the clinical features (Fig 2c) revealed that TAF exerted significantly superior efficacy to ETV in patients without underlying cirrhosis (P = 0.001), but not in those with underlying cirrhosis (P = 0.068). TAF was also found to be superior to ETV in both patients with genotype B HBV infection and genotype C HBV infection, regardless of the serum HBsAg and HBcrAg levels at the time of the drug switching, but only in patients who were seronegative for HBeAg. Chu CM *et al*. reported that HBsAg seroclearance showed correlation with the extent of hepatic steatosis [15]. However, in the present study, the extent of serum HBsAg level reduction during the TAF treatment period did not associate with serum levels of GGT and cholesterol as well as AST/ALT ratio at the during switching, suggesting that serum HBsAg level reduction did not associate with the extent of hepatic steatosis.

**Fig 3:**
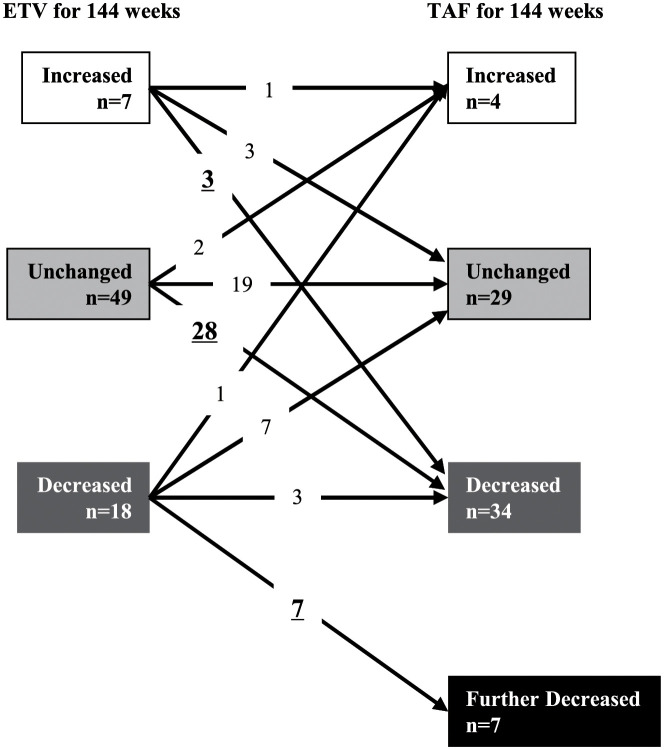
Changes in the serum HBsAg levels during the 48-week period of treatment with entecavir (ETV) prior to the drug switching and 48-week period of treatment with tenofovir alafenamide (TAF) after the drug switching. Increase and decrease of the serum HBsAg level by 0.24 Log IU/mL or more were defined as “increase” and “decrease,” respectively, and changes by <0.24 Log IU/mL were defined as “unchanged.” Further decrease of the level during the TAF treatment period by greater than 0.24 Log IU/mL in patients who showed “decrease” of the serum HBsAg level during the ETV treatment period was defined as “further decreased”.

When increase and decrease of the serum HBsAg level by ≥0.24 Log IU/mL during the 144-week treatment period was defined as “increased” and “decreased,” respectively, the serum HBsAg level increased in 7 patients (9.5%), remained unchanged in 49 patients (66.2%), and decreased in 18 patients (24.3%) during the ETV treatment period (Fig 3). Of the 7 patients in whom the serum HBsAg level increased during the ETV treatment period, it increased also during the TAF treatment period in 1 patient, but remained unchanged and decreased in 3 and 3 patients, respectively. In contrast, among the 49 patients in whom the serum HBsAg level remained unchanged during the ETV treatment period, the level continued to remain unchanged in 19 patients, increased in 2 patients, and decreased in 28 patients during the TAF treatment period. Moreover, among the 18 patients in whom the serum HBsAg level decreased during the ETV treatment period, the level increased and remained unchanged in 1 and 7 patients, respectively, while persistent decrease and further decrease were observed in 3 patients and 7 patients, respectively, during the TAF treatment period. Thus, 38 patients (51.4%) were classified as TAF responders, and evaluation by the McNemar test revealed superior therapeutic efficacy of TAF as compared to that of ETV (P = 0.003). Although there existed no association between the extent of reduction in serum HBsAg levels and those in serum ALT and AST levels both during the ETV treatment period and the TAF treatment period, serum AST levels at the time of the drug switching were higher in the TAF responders than in the remaining patients (18 vs. 15 U/L, P = 0.033), and the percentages of men and patients with underlying cirrhosis tended to be higher in the TAF responder group as compared to the remaining patients (P = 0.061 and P = 0.055, respectively). Multivariate analysis, however, failed to identify any of the aforementioned factors as exerting any significant influence.

### Antiviral efficacies of ETV and TAF based on the propensity score matching model

The effects of ETV and TAF on the serum HBsAg levels were evaluated following propensity score matching of the patients for demographic characteristics and clinical features in 67 of 74 patients at the 144-week period prior to the nucleos(t)ide analog switching from ETV to TAF and those in 67 of 74 patients at the time of the drug switching (Table 2). The serum HBsAg level decreased by a median (Log IU/mL/144 weeks) of -0.256 (IQR, -0.601 to -0.123) during the TAF treatment period, which was greater than the corresponding values of (-0.042; IQR, -0.216 to +0.124) during the ETV treatment period (P<0.001) (Fig 2d).

**Table 2:** Demographic characteristics and clinical features of 67 patients with chronic HBV infection following propensity score matching between the 144-week period before the nucleos(t)ide analog switching and at the time of the switching.

Factors	Median (Interquartile Range) or Number of Patients (%)		P-value
	ETV (n = 67)*	TAF (n = 67)*	
Age: years old	60 (49–66)	59 (49–67)	0.796
Men/Women	36/31	37/30	1.000
underling cirrhosis: present	5 (7.5)	5 (7.5)	1.000
Genotype A/B/C	1 (1.5): 17 (25.4): 49 (73.1)	2 (3.0): 17 (25.4): 48 (71.6)	0.842
HBsAg: IU/mL	1152.79 (486.23–2781.24)	1129.77 (515.53–2650.83)	0.805
HBeAg: positive	5 (7.5)	7 (10.4)	0.764
HBcrAg (Log U/mL) <3.0/≧3.0/indeterminate	24 (35.8)/37 (55.2)/6 (9.0)	25 (37.3)/37 (55.2)/5 (7.5)	0.946

* Demographic characteristics and clinical features of 67 patients 144 weeks before nucleos(t)ide analog switching and those of 67 patients at the switching are shown at ETV group and TAF group, respectively.

HBsAg, hepatitis B surface-antigen; HBeAg, hepatitis B e-antigen; HBcrAg, hepatitis B core-related-antigen.

### Safety after switching from ETV to TAF

No patient died and HCC did not develop in all patients during both the 144-week ETV treatment period and 144-week TAF treatment period. Moreover, TAF administration was discontinued in no patients due to severe adverse events.

**Fig 4:**
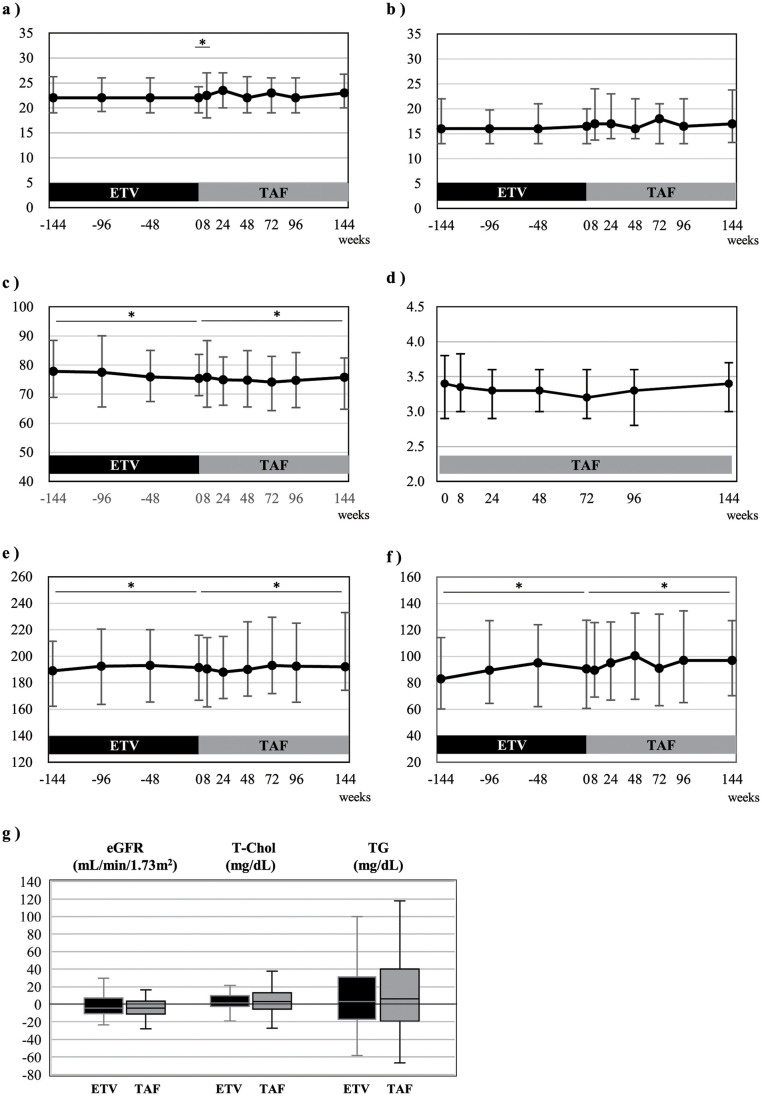
Laboratory tests in patients with chronic hepatitis B Virus (HBV) infection before and after switching of the nucleos(t)ide analog used for treatment from entecavir (ETV) to tenofovir alafenamide (TAF). a) serum alanine aminotransferase (ALT) levels, b) serum aspartate aminotransferase (AST) levels, c) estimate glomerular filtration rate (eGFR), d) serum inorganic phosphorus (IP) levels, e) serum total cholesterol levels, f) serum triglyceride levels; the points and error bars represent the median values and quartiles (Q1 and Q3), respectively. g) The box and whisker represent the quartiles (Q1 and Q3) and range, respectively. Changes in the serum total cholesterol and triglyceride levels during the 144-week ETV treatment period and 144-week TAF treatment period.

The median serum ALT level (U/L) increased from 16 (IQR, 13 to 20) to 17 (13 to 23) at 8 weeks after the drug switching (P = 0.002), but decreased to the baseline level by 24 weeks (P = 0.098 vs. the level at the time of switching), remaining unchanged thereafter until 144 weeks after the nucleos(t)ide analog switching (Fig 4a). Similar changes were observed for the serum AST level, although the differences were not statistically significant (Fig 4b). None of the patients developed serum ALT flares to over 5-fold of the upper limit of the standard values after the drug switching.

In regard to the renal safety, the eGFR (median; mL/min/1.73 m^2^) decreased significantly from 75.4 (IQR, 68.5 to 83.5) at the time of the nucleos(t)ide analog switching to 72.8 (65.1–83.8) at 48 weeks after the switching (P = 0.005), but remained unchanged thereafter until 144 weeks (Fig 4c). Similar decrease of the eGFR was observed during the 144-week ETV treatment period (from 77.8, 68.9–88.5 to 75.4, 69.5–83.6) (P = 0.034); the degree of decrease of the eGFR did not differ significantly during the ETV administration period and TAF administration period (-5.9% vs. -3.2%, P = 0.301). In contrast, the serum phosphorus levels remained unchanged during the 144-week TAF treatment period (Fig 4d).

The lipid profile was evaluated in 71 patients, after exclusion of 3 patients who were receiving lipid-lowering drugs at the timing of the drug switching. Both the median values (IQR) of the serum total cholesterol and serum triglyceride (mg/dL) increased, during the 144-week ETV treatment period as well as the 144-week TAF treatment period; the serum total cholesterol level increased from 189 (162–211) at 144 weeks prior to the drug switching to 192 (167–216) at the time of the drug switching (P = 0.038), and even further to 192 (174–233) at 144 weeks after the drug switching (P = 0.047) (Fig 4e); the serum triglyceride level increased from 83 (60–114) at 144 weeks before the drug switching to 91 (61–127) at the time of the drug switching (P = 0.044) and further to 97 (70–127) at 144 weeks after the drug switching (P = 0.012) (Fig 4f). The degrees of increase of the serum total cholesterol and triglyceride levels were, however, not different between the 144-week ETV treatment period and 144-week TAF treatment period; the percent increases relative to the baseline levels were +2.6% (IQR, -2.5 to +10.6) and +2.6% (-5.5 to +12.2), respectively, for the serum total cholesterol level (P = 0.678), and 0% (-21.3 to +26.2) and +6.1% (-19.1 to +40.9), respectively, for the serum triglyceride level (P = 0.477) (Fig 4g). None of the patients developed any cardiovascular events and none of the patients required initiation of a lipid-lowering drugs after the switching of ETV to TAF.

## Discussion

In our previous study, in which the extent of reduction of the serum HBsAg level between the 48-week periods before and after switching of the nucleos(t)ide analog used for treatment from ETV to TAF were compared in 92 patients with chronic HBV infection, the extent of reduction, overall, was greater during the 48-week TAF treatment period after the drug switching than during the 48-week ETV treatment period prior to the drug switching (Log IU/mL; 0.068 vs. 0.041), although the difference was not significant (P = 0.069); the extent of reduction during the TAF treatment period was, however, significantly greater in patients without underlying cirrhosis, patients with genotype B HBV infection, and patients with serum HBcrAg levels of <3.0 Log U/mL [11]. Moreover, the McNemar test revealed that the antiviral efficacy of TAF was superior to that of ETV when increase and decrease of the serum HBsAg level by 0.08 Log IU/mL/48 weeks or more were defined as “increase” and “decrease,” respectively (P = 0.022) [11]. Controversy exists, however, as to whether TAF exerts superior efficacy to ETV in reducing the serum HBsAg level [16–21]. Kumada T, *et al*. reported results similar to ours [11] in patients in whom the nucleos(t)ide analog used for treatment was switched from ETV to TAF [16]; on the other hand, Ogawa E, *et al*. [17] did not find any superiority of TAF over ETV, in terms of the efficacy of the drugs in reducing the serum HBsAg levels. Moreover, Hagiwara *et al*. reported similar degrees of reduction of the serum HBsAg level between patients in whom the nucleos(t)de analog was switched from ETV to TAF and those who had received ETV monotherapy [18,19]. Similar results were also reported by Itokawa *et al*. [20], Li ZB *et al*. [21], and Tamaki N, *et al*. [22]. There were limitations of these studies, e.g., small study sample sizes [18,19,21,22], short TAF administration period (24 weeks) [21], and different sites of measurement of the serum HBsAg levels [17,20]. Thus, in the present study, we compared the degrees of reduction of the serum HBsAg levels over the long term (144 weeks) in the same cohort of patients as in our previous study [11], by comparison of the level between the 144-week ETV treatment period prior to switching of the nucleos(t)de analog from ETV to TAF and the 144-week TAF treatment period after the drug switching. The evaluation was conducted exclusively in patients who had received antiviral therapy with ETV for at least for 192 weeks prior to the drug switching, in order to compare the degrees of reduction of the serum HBsAg levels under the condition of suppressed HBV genome replication, since the amount of covalently closed circular (ccc)DNA in the hepatocytes may affect the serum HBsAg levels.

Consequently, the evaluation was conducted in 74 of the 92 patients enrolled in the previous study [11], and the extent of reduction of the serum HBsAg level was shown to be greater during the 144-week TAF treatment period after the drug switching than during the 144-week ETV treatment period prior to the drug switching (Log IU/mL; 0.252 vs. 0.046, P<0.001). Significant differences in the degree of reduction of the serum HBsAg levels were seen in patients with genotype C HBV infection as well as those with genotype B HBV infection, irrespective of the serum HBsAg and HBcrAg levels at the time of switching of the nucleos(t)ide analog. Analysis by the McNemar test revealed that when increase and decrease of the level by 0.24 Log IU/mL/144 weeks or more were defined as “increase” and “decrease,” respectively, TAF was found to exert superior efficacy as compared to ETV (P = 0.003) in reducing the serum HBsAg level.

We were afraid that the extent of serum HBsAg level reduction might be accelerated along with the elongation of nucleos(t)ide analog treatment periods probably due to aging of patients and decreased serum HBsAg level at the late phase of the treatment periods. Thus, in the present study, the effects of ETV and TAF on the serum HBsAg levels were evaluated in following propensity score matching of the patients for demographic characteristics and clinical features, including the age and serum HBsAg levels, between 67 patients selected from a total of 74 patients at the 144-week period prior to the nucleos(t)ide analog switching from ETV to TAF and the different set of 67 patients at the time of the drug switching. The results of this analysis conducted after propensity score matching also revealed the superiority of TAF over ETV in terms of the efficacy of reducing the serum HBsAg levels (P<0.001).

The mechanisms involved in the superiority of TAF over ETV in serum HBsAg reduction are to be clarified. Recently, however, Murata *et al*. reported that nucleotide analogs, such as ADV and TDF, directly induced IFN-λ3 in the intestinal mucosal cell leading to reduction in HBsAg production in hepatocytes in patients with HBV infection [23]. Moreover, they showed that TDF, but not ETV inhibited lipopolysaccharide mediated IL-10 production, while increased IL-12p70 and TNF-α production in peripheral blood mononuclear cells, and consequently TDF reduced HBs antigen production in hepatocytes in a dose-dependent manner, resulting in the lowering effect of HBs antigen in hepatocytes [24]. Similar mechanisms may be involved in serum HBsAg reduction in patients receiving TAF, and should be clarified in future.

Moreover, there were no adverse events encountered during the 144-week TAF treatment period in the present cohort. In our previous 48-week outcome study mentioned above, we reported the absence of any serious safety concerns during the TAF treatment period, except for transient and slight increase of the serum AST and ALT levels at 8 and 24 weeks after the switching of the nucleos(t)ide analog [11]. There were no cases of decline of the eGFR or unfavorable changes of the serum inorganic phosphorus or serum AST and ALT levels during the TAF administration period from 48 to 144 weeks after the drug switching. In the present study, the effects of TAF and ETV on the serum lipid profiles were also compared, since the serum total cholesterol levels were reported, by previous studies, to increase following switching of the nucleos(t)ide analog from TDF to TAF in patients with HIV infection [25,26]. Recently, Suzuki K, *et al*. reported that TDF decreased the serum total cholesterol level in patients with chronic HBV infection through CD36/peroxisome proliferator-activated receptor (PPAR)-α activation in the hepatocytes [27]. In the present study, the serum total cholesterol and serum triglyceride levels increased during both the 144-week ETV treatment period and 144-week TAF treatment period, probably due to the advancing age of the patients, and the degrees of increase were similar between the two periods, suggesting that TAF may not affect lipid metabolism in the hepatocytes unlike TDF, and the impact of TAF on the risk of cardiovascular events was similar to that of ETV in patients with chronic HBV infection. Moreover, Surial B, *et al*. reported that body weights of patients were increased after the switching nucleotide analog from TDF to TAF in patient with HIV [28]. In the present study, however, the serial changes in body weight of patients were not evaluated because of the retrospective study design. These matters should be investigated in future.

The limitation of the present study is absence of the control arm, in which patients received ETV continuously. The limitation existed also in the propensity score matching analysis conducted to exclude the effect of aging of patients and decrease of serum HBsAg levels on serum HBsAg level reduction. In the present study, a set of 67 selected from 74 patients at 144 weeks before the switching of nucleos(t)de analog and an another set of 67 patients selected from 74 patients at the switching of nucleos(t)de analog were compared. The number of patients for the propensity score matching were almost similar to the total number of patients. Moreover, total treatment periods using nucleos(t)ide analogs could not be matched in the propensity score matching analysis according to the present study design. Thus, the prospective trials comparing the serum HBsAg level reduction between patients receiving ETV continuously and those receiving switching nucleos(y)ide analog from ETV to TAF should be done in the future. Furthermore, the extents of serum HBsAg level reduction were less than 1.0 Log IU/mL in the most patients receiving TAF for 144 weeks and serum HBsAg became undetectable in none of the patients. Yip *et al*. reported, however, that TDF was superior to ETV in prevention of HCC in patients with chronic HBV infection [29]. Thus, the effects of switching of nucelos(t)ide analog from ETV to TAF on hepatocarcinogenesis are to be elucidated in the future.

In conclusion, switching of the nucleos(t)ide analog used for treatment from ETV to TAF merits consideration in patients with chronic HBV infection who are receiving ETV monotherapy, since TAF was found to exert superior efficacy to ETV in reducing the serum HBsAg level in both patients with genotype C HBV infection and patients with genotype B HBV infection, irrespective of the serum HBsAg and HBcrAg levels at time of the drug switching. The mechanisms involved in the reduction of the serum HBsAg level in patients receiving TAF needs to be further evaluated. The impact of reduction of the serum HBsAg level by TAF on the risk of development of HCC in patients with chronic HBV infection should also be examined in comparison with that by ETV.

## Supporting information

S1 Checklist TREND statement checklist. (PDF)

S1 File (PDF)

S2 File (PDF)

S1 Dataset (XLSX)
