# Supplementary material for: Superiority of tenofovir alafenamide fumarate over entecavir for serum HBsAg level reduction in patients with chronic HBV infection: A 144-week outcome study after switching of the nucleos(t)ide analog
Source: PLoS One. 2022 Feb 18;17(2):e0262764. doi: 10.1371/journal.pone.0262764 (PMC8856517; doi:10.1371/journal.pone.0262764)
Supplement: S1 File — (PDF) [file pone.0262764.s002.pdf]

Evaluation of the efficacy of switching to tenofovir alafenamide fumarate in patients with chronic liver disease type B receiving nucleic acid analogues

Principal Investigator
<Research Institution> Saitama Medical University Hospital
＜Affiliation(s): Department of Gastroenterology and Hepatology
＜Satoshi Mochida

1. Purpose of the study

To evaluate the long-term efficacy and safety of switching from nucleic acid analogues (NA) to tenofovir alafenamide fumarate (TAF) in patients with chronic liver disease type B who are taking NA.

2. Background and significance of the study

About 400 million people worldwide are persistently infected with hepatitis B virus (HBV), and some of them develop liver cirrhosis and hepatocellular carcinoma as a result of persistent chronic hepatitis, which is a stage of immune elimination. In Japan, about 30,000 people die annually from hepatocellular carcinoma, and HBV infection accounts for about 15% of these deaths. On the other hand, HBV carriers may have acute exacerbations, and HBV infection is responsible for about 40% of acute and delayed liver failure in Japan.

In Japan, four NA drugs, lamivudine (LAM), adefovir pivoxil (ADV), entecavir hydrate (ETV), and tenofovir disoproxil fumarate (TDF), as well as interferon (IFN) and peginterferon (Peg-IFN The administration of NA lowers the HBV-DNA level and suppresses the progression to cirrhosis by subsiding hepatitis, but hepatocarcinogenesis cannot be suppressed unless the HBs antigen level is reduced.

In December 2016, TAF was approved as the fifth NAF for chronic liver disease type B. The drug has been positioned as the first choice for HBV treatment in the Japanese Society of Hepatology's "Guidelines for Hepatitis B Treatment," not only for HBV suppression but also for HBs antigen reduction, lack of emergence of drug-resistant mutations, and safety, including renal protection. However, there is no evidence of long-term treatment with TAF after switching from NA to TAF in patients receiving existing NA such as ETV, and the effect of TAF on changes in HBs antigen levels is unknown.

In this study, we prospectively evaluated the long-term effects on HBs antigen levels and safety of switching from NA to TAF to reduce HBs antigen levels in patients with chronic liver disease type B who were receiving NA for at least 1 year and who were attending the outpatient clinic of the Department of Gastroenterology and Hepatology, Saitama Medical University Hospital. The long-term effects on HBs antigen levels and safety will be prospectively investigated.

In this study, reduction of HBs antigen levels in patients with type B liver disease will enable us to discontinue NA administration, which will not only reduce patient burden but also reduce medical costs. In addition, the decrease in HBV activity is expected to reduce the risk of future liver carcinogenesis.

3. Organization of the study

1) Principal Investigator
Affiliation: Department of Gastroenterology and Hepatology
Position: Professor
Name: Satoshi Mochida

2) Principal investigator: See Appendix
3) Collaborating institutions and principal investigators (e.g., multi-institutional collaborative research, if applicable): Not applicable
4) Research secretariat and person in charge (if applicable): Not applicable
5) External organizations other than the above, such as data centers (if applicable): Not applicable

4. Method and duration of the research
1) Design
Prospective interventional observational study
2) Criteria for enrollment/exclusion of study subjects
Enrollment criteria:
(1) Chronic hepatitis B patients who have been taking ETV or TDF/ADV/LAM for at least 1 year
(2) HBs antigen level of 100 IU/mL or higher
(3) Age 20 years or older
(4) HBeAg positive or not, and on IFN or not.

Exclusion criteria
(1) Patients receiving immunosuppressive drugs
(2) Patients with HIV co-infection
(3) Patients receiving contraindications listed in the package insert.
(3) Patients who are receiving contraindicated drugs in the package insert (4) Other patients who are deemed inappropriate to participate in the study by the physician in charge

3) Target number of patients
Total number of cases: 200 cases
Number of cases at Saitama Medical University Hospital 200 cases
4) Period of the study
Enrollment period: Approval date - March 31, 2019
Research period: Approval date - March 31, 2025

5) Schedule
TAF (Bemlidy®) 25 mg/day (regular dose) will be administered orally at outpatient clinic after obtaining consent for the clinical study.
6) Observation items/investigations/test items
Primary endpoints
To compare the changes in HBs antigen levels and the frequency of adverse events (including abnormal laboratory values and other laboratory abnormalities) during the 1 year before and 5 years after the start of TAF.
Secondary endpoints
(1) Changes in HBs antigen levels after 1, 2, 3, and 4 years of treatment
(1) Changes in HBs antigen levels after 1, 2, 3, and 4 years of treatment (2) Occurrence of adverse events (including abnormal laboratory values and other laboratory abnormalities) after 1, 2, 3, and 4 years of treatment
(3) Changes in clinical laboratory values: WBC, WBC fraction, Hb, Plt, AST, ALT, γ-GTP, ALP, LDH, BUN, T-Bil, TP, Alb, Cre, BUN, eGFR, Na, K, Cl, IP, Ca, AFP, HBV-DNA, HBe antigen, HBe antibody, HBcr antigen, urinary protein Urinary protein, urinary NAG, urinary Cre, urinary IP
(4) Frequency of new hepatocarcinogenesis in the first 5 years after initiation of treatment

7) Restrictions on concomitant therapy and concomitant drugs
None
8) Discontinuation criteria
When serious complications occur.
When oral administration becomes impossible.
9) Method of statistical processing
The results obtained will be statistically processed on the PC in the Gastroenterology and Hepatology Laboratory.
10) Others
None 5.

5. Disclosure of information about the study

Registration in the database at the beginning of the study (required for interventional studies)
Name of the database: UMIN-CTR
Registration number: UMIN000030661

6. Storage of samples and information

The physician in charge of the study will conduct the stipulated examinations and observations during the study period and obtain the necessary data. A case report form will be written for all enrolled cases, including those who have discontinued the study treatment. The case report should include the details of the treatment administered during the study treatment and observation periods, the results of observations and examinations, and adverse events. The items to be included in the case report form are the items of information to be collected.

The results and other ancillary information obtained in this study will be kept in the laboratory of the Department of Gastroenterology and Hepatology, Saitama Medical School until five years after the completion of the study or three years after the final publication of the study results, whichever is later. After that, the information will be disposed of appropriately to prevent leakage of personal information.

In addition, the results obtained in this study may be used in other studies for future research, and in such cases, the results will be used only after obtaining another approval from the hospital IRB. This will be clearly stated in the explanatory document.

7. Methods of protecting personal information

Since this is a clinical research conducted solely by the hospital and all data analysis, etc., will be conducted at the hospital, personal information such as names, IDs, and dates of birth of the subjects will not be disclosed during the research. We will also give due consideration to the protection of the personal information of the test subjects at the time of publication.

Personal information manager of this hospital in this study
Name of personal information manager: Miyuki Yagi Affiliation: Gastroenterology and Hepatology Position: Secretary

8. Informed consent

The principal investigator will prepare a document explaining this research to the subjects. The principal investigator and sub-investigators shall provide sufficient explanation using the explanatory document to the subjects who are scheduled to participate in the study, and confirm that they have given their written consent of their own free will after fully understanding the participation in the study.

If the content of the explanatory document changes, the latest version should be used to obtain informed consent.

9. Benefits and Disadvantages

1) Benefit (expected effect): Reduction of HBs antigen levels will enable discontinuation of NA administration; achieving drug free in patients with chronic liver disease type B will not only reduce patient burden but also reduce medical costs. In addition, the benefits to patients are substantial, as they are expected to reduce the risk of future hepatocarcinogenesis. In addition, there is no need to reduce the dosage in patients with renal dysfunction, and continuous treatment is possible safely.

2) Disadvantages (possible adverse reactions): In the results of the international Phase III study up to 48 weeks, adverse reactions including abnormal laboratory values were observed in 123 (14.2%) of 866 patients with chronic liver disease type B (including 56 Japanese patients). The major adverse reactions included nausea in 17 cases (2.0%), fatigue and headache in 12 cases (1.4%) each, and abdominal distension in 9 cases (1.0%), none of which were serious.

3) Methods to minimize risk (disadvantage): Discontinuation criteria should be established.

10. Endpoints

To compare the trend of decrease in HBs antigen levels and the frequency of adverse events (including abnormal laboratory values and other laboratory abnormalities) during the first year before and the first 5 years after TAF administration.

11. Compensation details in the event of health damage to subjects

In the event that an adverse event occurs as a result of the conduct of the study, appropriate measures will be taken immediately to ensure that the subject receives appropriate treatment. Since the subjects participating in this study are being treated within the scope of insurance coverage, the "Pharmaceutical Adverse Drug Reaction Relief System" will be applied in the event of the occurrence of unpredictable serious adverse drug reactions.

12. Response to consultation from subjects regarding the research

The following is the contact information for consultation.

Contact: Department of Gastroenterology and Hepatology, Saitama Medical University Hospital

Daytime (9:00-17:00): Outpatient Department of Gastroenterology and Hepatology, Saitama Medical University Hospital 049-276-1279

Nighttime (5:00-9:00 p.m.): Ward, Department of Gastroenterology and Hepatology, Saitama Medical University Hospital 049-276-1320

13. Matters related to expenses

1) Source of funding for the study

All costs for this study will be borne by the Department of Gastroenterology and Hepatology, Saitama Medical University.

2) Conflict of interest in the research of the research institution

The Department of Gastroenterology and Hepatology has an industry-university relationship with Gilead Sciences Corporation, the distributor of the drug in this study, but this does not affect the results of this study.

3) Economic burden on the subjects

This study is covered by insurance, and there will be no financial burden to the subjects other than the cost of medical examination.

4) Gratuity to subjects

None.

14. Matters related to reporting to the hospital director

1) Permission to Conduct Research

The principal investigator shall confirm that the research protocol has been approved by the hospital IRB and authorized by the hospital director prior to the implementation of the research.

2) Changes to the research protocol: The principal investigator shall confirm that the research protocol has been approved by the hospital IRB and authorized by the hospital director prior to the implementation of the research.

If any changes occur in the contents of the research protocol or the consent/explanation documents, the principal investigator shall promptly apply for changes to the hospital director, obtain the approval of the hospital IRB, and obtain the approval of the hospital director.

3) Implementation status report

The principal investigator will report the status of the research to the hospital director and the hospital IRB at least once a year.

4) At the end of the research

The principal investigator will report to the hospital director and the hospital IRB as soon as the research is completed.

5) Response to the occurrence of serious adverse events (only in the case of invasive procedures)

If a serious adverse event occurs in the course of conducting the research, the principal investigator should immediately take necessary measures and promptly report to the hospital director.

15. Monitoring and auditing (with invasion and intervention)

In accordance with the "Monitoring and Auditing Procedures for Saitama Medical University Hospital", monitoring by a third party will be conducted for this study because 200 cases are planned, although there is no random assignment within the medical insurance indications. There is no need for an audit.

Person in charge of monitoring (Clinical Research Management Center)

Person in charge of audit (Not applicable)

16. Matters related to genetic testing (only if applicable)

Not applicable.

17. Publication of research results

The results will be published in UMIN-ICR and presented at academic meetings such as the Japanese Association for the Study of the Liver, the American Association for the Study of Liver Diseases, the European Association for the Study of Liver Diseases, and the Asia Pacific Rim Liver Conference. In addition, we will prepare and present papers in English.
