# Supplementary material for: Superiority of tenofovir alafenamide fumarate over entecavir for serum HBsAg level reduction in patients with chronic HBV infection: A 144-week outcome study after switching of the nucleos(t)ide analog
Source: PLoS One. 2022 Feb 18;17(2):e0262764. doi: 10.1371/journal.pone.0262764 (PMC8856517; doi:10.1371/journal.pone.0262764)
Supplement: S2 File — (PDF) [file pone.0262764.s003.pdf]

核酸アナログ製剤投与中 B 型慢性肝疾患患者におけるテノホビルアラフェナミドフマル酸塩へ切り替えの有用性の検討
研究計画書

研究責任者
＜研究機関名＞埼玉医科大学病院
＜所属＞　消化器内科・肝臓内科
＜氏名＞　　　　　　　持田　智

１． 研究の目的

核酸アナログ製剤（NA）を内服している B 型慢性肝疾患患者において，NA をテノホビルアラフェナミドフマル酸（TAF）に切り替えることの長期的な有用性と安全性を検討する。

２． 研究の背景と意義

B 型肝炎ウイルス（HBV）は，全世界で約 4 億人の持続感染者が存在し，その一部は免疫排除期である慢性肝炎の状態が持続して肝硬変へ進展，肝細胞癌を併発する。わが国では年間約 3 万人が肝細胞癌で死亡するが，その成因は HBV 感染が約 15%を占めている。一方，HBV キャリアは急性増悪を来たす場合もあり，我が国の急性肝不全および遅発性肝不全の成因は HBV 感染が約 40%を占めている。

我が国では，B 型慢性肝疾患に対する治療薬としてラミブジン（LAM），アデホビルピボキシル（ADV），エンテカビル水和物（ETV），テノホビルジソプロキシルフマル酸塩（TDF）の 4 種類の NA と，インターフェロン（IFN）およびペグインターフェロン（Peg-IFN）α2a が認可されている。NA の投与により HBV-DNA 量は低下し，肝炎が沈静化することで肝硬変への進展を抑制できるようになったが，HBs 抗原量が低下しないと肝発癌は抑制することができない。

2016 年 12 月に B 型慢性肝疾患に対する 5 つ目の NA として，TAF が承認された。同薬剤は HBV の鎮静化のみならず HBs 抗原量の低下効果，薬剤耐性変異の出現がないこと，および腎保護作用などの安全性の観点から，日本肝臓学会「B 型肝炎治療ガイドライン」において HBV 治療の第一選択として位置づけられている。しかし，ETV などの既存の NA を投与されている症例において，NA を TAF に切り替えた後の長期投与のエビデンスはなく，特に HBs 抗原量の変化に対する影響は不明である。

そこで埼玉医科大学病院消化器内科･肝臓内科の外来を通院中で NA を少なくとも 1 年以上投与されている B 型慢性肝疾患患者のうち，HBs 抗原量が 100 IU/mL 以上の症例を対象として，HBs 抗原量の低下を目的として NA を TAF に変更し，長期的な HBs 抗原量への影響および安全性について前向きに検討する。

本研究において B 型肝疾患患者の HBs 抗原量を減少することで，NA 投与を中止することが可能となり，患者負担の削減だけでなく医療費の削減にもつながる。さらに，HBV の活動性が低下することで将来の肝発がんのリスクを減少が予想される。

３．研究の実施体制・組織

１）主任研究者

所属　消化器内科・肝臓内科　　（役職　教授）　　氏名　持田　智

２）研究実施者：別紙記載

３）共同研究機関及び研究責任者（多施設共同研究など、該当する場合）：該当なし

４）研究事務局及び担当者（該当する場合）：該当なし

５）上記以外のデータセンターなどの外部機関（該当する場合）：該当なし

４．研究の方法と期間

１）デザイン

前向き介入観察研究

２）研究対象者の登録基準/除外基準

登録基準：

（１）ETV もしくは TDF・ADV・LAM を 1 年間以上内服中の慢性 B 型肝疾患

（２）HBs 抗原量が 100 IU/mL 以上

（３）20 歳以上

（４）HBe 抗原陽性および，IFN 投与中の有無は問わない

除外基準：

（１）免疫抑制剤の投与を受けている患者

（２）HIV に重複感染している患者

（３）添付文書にある併用禁忌の薬剤を投与中の患者

（４）その他，担当医師が本試験への参加が不適切と判断した患者

３）目標症例数

全体症例数　200 例

埼玉医科大学病院における症例数　200 例

４）研究の期間

登録期間：承認日～2019 年 3 月 31 日

研究期間：承認日～2025 年 3 月 31 日

５）スケジュール

臨床研究の同意取得後より，外来にて TAF （ベムリディ®）25mg/day（常用量）の経口投与を行う。

６）観察項目/調査項目/検査項目

・主要評価項目

TAF 投与開始前 1 年間と，投与開始後 5 年間の HBs 抗原量の推移および有害事象（臨床検査値異常およびその他の検査異常を含む）の発生頻度を比較する。

・副次的評価項目

（１）投与開始 1，2，3，4 年後の HBs 抗原量の推移

（２）投与開始 1，2，3，4 年後の有害事象（臨床検査値異常およびその他の検査異常を含む）の発生

（３）臨床検査値の推移：WBC，WBC 分画，Hb，Plt，AST，ALT，γ-GTP，ALP，LDH，

BUN，T-Bil，TP，Alb，Cre，BUN，eGFR，Na，K，Cl，IP，Ca，AFP，HBV-DNA，HBe 抗原，HBe 抗体，HBcr 抗原，尿中蛋白，尿中 NAG，尿中 Cre，尿中 IP
（４）投与開始後 5 年間の新規肝発癌の頻度
７）併用療法や併用薬剤の制限
なし
８）中止基準
重篤な合併症が出現したとき。
経口投与が不可能となったとき。
９）統計処理を行う方法
得られた成績は消化器内科・肝臓内科研究室の PC にて統計処理を行う。
１０）その他
なし

５．研究に関する情報公開について

研究開始時のデータベースへの登録（介入研究は必須）
データベースの名称：UMIN-CTR
登録番号：UMIN000030661

６．試料・情報の保管について

担当医師は研究期間中、規定された検査、観察を行い、必要なデータを取得する。試験治療を中止した症例も含め、登録した全ての症例について症例報告書を記載する。症例報告書には試験治療期間および観察期間の実施治療内容、観察・検査の結果、有害事象などを記入する。症例報告書に記載するべき事項が、収集されるべき情報の項目となる。

本研究で得られた成績やその他の付帯情報は研究終了から 5 年，ないし研究結果の最終の公表から 3 年のどちらか遅い期日まで，埼玉医科大学消化器内科・肝臓内科の研究室にて保管する。その後，個人情報の漏洩に配慮し適切に廃棄する。

また，本研究で得られた成績は将来の研究のために他の研究で使用される可能性があり，その際には改めて病院 IRB で承認を得た上で使用する。このことは説明文書に明記する。

７．個人情報保護の方法

当院単独の臨床研究であり、データの解析等もすべて当院で実施するため、研究実施中に被験者の氏名、ID、生年月日などの個人情報が、外部に出ることはない。公表時にも被検者の個人情報保護については十分に配慮する。

本研究における当院の個人情報管理者
個人情報管理者氏名：八木美由紀　所属：消化器内科・肝臓内科　役職：秘書

８．インフォームドコンセント

研究責任医師は、本研究に関する被験者への説明文書を作成する。研究責任医師及び分担医師は、当該研究への参加を予定している被験者に対して、説明文書を用いて十分に説明し、研究の参加について十分に理解したのち、自由な意思により、文書にて同意を得たことを確認する。

説明文書の内容が変更した際には、最新版を用いてインフォームドコンセントを受けることとする。

９．利益と不利益

１）利益（期待される効果）: HBs 抗原量を減少させることで，NA 投与の中止が可能となる。B 型慢性肝疾患患者における drug free を達成することで，患者負担の削減だけでなく，医療費の削減が可能である。さらに，将来の肝発がんのリスクを減少が予想され，患者の得られる利益は多大である。また，腎機能障害の症例などにおいて減量投与の必要がなく，安全に継続的な治療が可能となる。

２）不利益（起こりうる副作用）: 国際共同第III相試験における 48 週時までの成績では，B 型慢性肝疾患患者 866 例（日本人 56 例を含む）中 123 例（14.2%）に臨床検査値異常を含む副作用が認められた。主な副作用として，悪心 17 例（2.0%），疲労及び頭痛各 12 例（1.4%），腹部膨満 9 例（1.0%）が挙げられるが，いずれも重篤ではない。

３）リスク（不利益）を最小化する方法：中止基準を設ける。

１０．評価項目（エンドポイント）

TAF 投与開始前 1 年間と，投与開始後 5 年間の HBs 抗原量の低下の推移および有害事象（臨床検査値異常およびその他の検査異常を含む）の発生頻度を比較する。

１１．被験者に健康被害が生じた場合の補償内容

研究の実施に伴い、有害事象が発現した場合は、ただちに適切な処置が受けられるよう対応する。本研究に参加した被験者は、保険適応内での治療を行っていることから、予測できない重篤な副作用発現時には、「医薬品副作用被害救済制度」が適応される。

１２．研究に関する被験者からの相談等の対応

以下の連絡先を相談窓口とする。

連絡先：埼玉医科大学病院　消化器内科・肝臓内科

昼間（9:00～17:00）: 埼玉医科大学病院　消化器内科・肝臓内科外来　049-276-1279

夜間（17:00～9:00）: 埼玉医科大学病院　消化器内科・肝臓内科病棟　049-276-1320

１３．費用に関する事項

１）研究の資金源

本研究に要する費用は，全て埼玉医科大学消化器内科・肝臓内科で負担する。

２）研究機関の研究に係る利益相反について

消化器内科・肝臓内科は本研究における対象薬剤販売元であるギリアド・サイエンシズ株式会社との間に，産学連携関係が存在しているが，これが本研究の結果に影響することはない。

３）被験者への経済的負担

本研究は保険診療内であり，受診に関する費用以外に被験者への経済的負担は発生しない。

４）被験者への謝礼

なし。

１４．病院長への報告に関する事項

１）研究の実施の許可：

研究責任者は，研究の実施に先立ち本研究計画書について病院ＩＲＢの承認及び病院長の許可を得ていることを確認する。

２）研究計画内容の変更：

研究責任者は，研究計画書内容や同意文書・説明文書内容に変更点が生じた場合は速やかに病院長に変更申請をし，病院ＩＲＢの承認を得て病院長の許可を得る。

３）実施状況報告：

研究責任者は，少なくとも年に１回以上の頻度で研究の実施状況を病院長及び病院ＩＲＢに報告する。

４）研究終了時：

研究責任者は，研究が終了したら速やかに病院長と病院ＩＲＢに報告をする。

５）重篤な有害事象発現時の対応（侵襲ありの場合のみ）：

研究責任者は，研究の実施に伴い，重篤な有害事象が発現した場合は，ただちに必要な処置を講ずるとともに、速やかに病院長に報告する。

１５．モニタリング・監査について（侵襲あり・介入ありの場合）

本研究の実施にあたり「埼玉医科大学病院モニタリング監査手順書」に従い，医療保険適応内で無作為割付けがないが，200 例の症例を予定しているため，第 3 者によるモニタリングを実施する。監査の必要はない。

モニタリング担当者　　（臨床研究管理センター）

監査担当者　　　　　　（該当なし）

１６．遺伝子検査に関する事項　（該当する場合のみ）

該当なし。

１７．研究結果の公表

UMIN-ICR で公表するとともに，日本肝臓学会，米国肝臓病会議，欧州肝臓病会議，アジア環太平洋肝臓会議など学術集会で発表する。さらに，英文論文を作成して発表する。
